# Baseline and follow-up assessment of regional left ventricular volume using 3-dimensional echocardiography: comparison with cardiac magnetic resonance

**DOI:** 10.1186/1476-7120-7-55

**Published:** 2009-11-19

**Authors:** Carly Jenkins, Thomas H Marwick

**Affiliations:** 1 Department of Medicine, University of Queensland, Brisbane, Australia

## Abstract

The assessment of regional volumes is an option for analysis of the response of LV segments to interventions such as revascularization or cell therapy. We sought to compare regional volumes from 3D-echocardiography (3DE) with cardiac magnetic resonance (CMR) over follow-up.

CMR regional volumes were assessed at baseline and after one year follow-up in 30 unselected patients (28 men, 65 ± 11 years) presenting for evaluation of cardiac function with previous infarction. 3DE images were also gathered over 4 cardiac cycles and measurements were performed off-line. CMR images were obtained using a 1.5 Tesla scanner and measured offline by method of landmarks and by centre of mass. Regional volumes were measured at end-diastole (rEDV) and end-systole (rESV) and the change in volume was compared for each over follow-up.

There was good correlation between 3DE and both CMR methods at baseline and follow-up. Changes in rEDV with 3DE vs CMR_L _were comparable (0.11 ± 3 ml vs 0.12 ± 3 ml, p = 0.94), as was change in CMR_M _(0.26 ± 2 ml, p = 0.69). However the change in regional volume by 3DE and CMR_L _correlated poorly (r = 0.03, p = 0.68), as did change in 3DE vs CMR_M _(r = 0.04, p = 0.65). Similarly, changes in rESV with 3DE and CMR_L _were similar (0.27 ± 2 ml vs 0.36 ± 2 ml, p = 0.70), as was change in CMR_M _(0.05 ± 1 ml, p = 0.31). Again, correlations between rESV by 3DE vs CMR_L _were poor (r = 0.03, p = 0.72), as well as 3DE vs CMR_M _(r = 0.07, p = 0.40).

Although global 3DE volumes compare well with CMR volumes, new developments in image quality and automated software will be needed before changes in regional volumes can be reliably followed with 3DE.

## 

The assessment of regional LV volumes may permit the analysis of the response of LV segments to interventions such as resynchronisation therapy [1]. The technical details of regional volume measurement are critically important. Use of a fixed external frame of reference in analyses of regional wall motion in the apical four-chamber view is prone to a systematic error [2]. Both cardiac magnetic resonance (CMR) and 3-dimensional echocardiography (3DE) perform regional analysis after first identifying important landmarks such as the LV apex, aortic valve and mitral annulus and RV insertions [3]. Use of a floating-axis analysis avoids the systematic error associated with translation around a fixed axis, but this is based on landmarks by CMR (apex, annulus)[4] and center of mass by 3DE. These axes may be influenced by reverse remodeling after intervention. Although *global *3DE volumes compare well with CMR volumes over time [5], the evolution of *regional *volumes over time is yet to be investigated. Encouraging data have been reported with the cross-sectional comparison of 3DE and CMR [6], but the ability to sequentially follow regional volumes with 3DE is undefined. We sought to compare regional volume assessment by 3DE and CMR over follow-up.

## Methods

### Study design

We prospectively recruited 30 patients with a history of prior infarction, who presented to the clinical laboratory for evaluation of LV parameters with 3DE and CMR. Contrast agents were not used in this study. The investigations were approved by the ethics committee of the Princess Alexandra Hospital, and all patients gave informed consent.

### 3D Echocardiography Acquisition

3DE images were obtained from an apical window with the patient in the left lateral decubitus position. Full-volume images were gathered over 4 cardiac cycles using a matrix array transducer (×4 transducer, Philips Sonos 7500 system). Only patients with interpretable images were included (eg. studies with stitch artifacts were excluded).

### 3D Echocardiography Measurement

**Figure 1:**
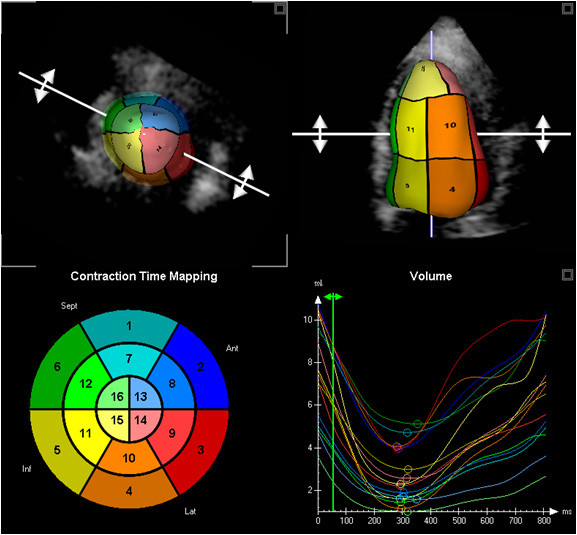
**Regional 3DE measurement; 16 segment model (bottom left) with corresponding LV shape (top images) and time volume curves of all 16 segments (bottom right)**.

Measurements of 3DE volumes were performed off-line using semi-automated border detection software (4D analysis CAP, Tomtec Gmbh, Unterschlessheim, Germany). The apical 4- chamber is used as the reference plane, with the apical 2- and 3-chamber views derived automatically from a 60 degree rotation between planes, using manual adjustments as required. Frames for EDV and ESV measurement were identified in accordance with American Society of Echocardiography guidelines, [7] EDV measurements at the frame following mitral valve closure and ESV measured on the image with the smallest left ventricular cavity. Initial contours were set by tracing the endocardial borders end-diastolic and -systolic images in the apical views. Contour tracing was performed with automatic border detection and manual editing used as required per step in the cardiac cycle and in a 3D model. Regional volumes (rEDV and rESV) were measured from the resulting 3D volume (Figure 1).

### MRI Acquisition

Cardiac magnetic resonance images were obtained using a Sonata 1.5 Tesla scanner (Siemens, Erlangen, Germany). Left ventricular anatomy and functional images were acquired in horizontal and vertical long axis views and short axis views using free induction, steady state precession imaging during a breath hold.

### MRI Measurement by Landmarks Method (CMR_L_)

**Figure 2:**
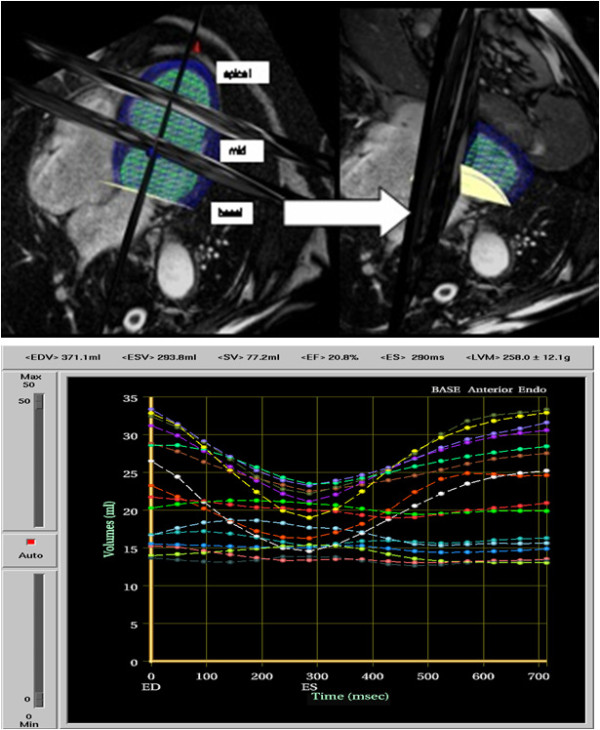
**Regional Volume measured by CMR_L; _Matrix of LV shape (top image) and corresponding time volume curves of all 16 segments (bottom image)**.

Offline calculation of the rEDV and rESV was performed using offline semi-automated border detection software (Cardiac Image Modeling version 4.2, Auckland University). Using two long axis and six or more short axis views, EDV and ESV were identified by the same method as 3DE, markers were placed on the right ventricle and left ventricular annulus, and the endocardial border was detected automatically (Figure 2).

### MRI Measurement by Centre of Mass Method (CMR_M_)

**Figure 3:**
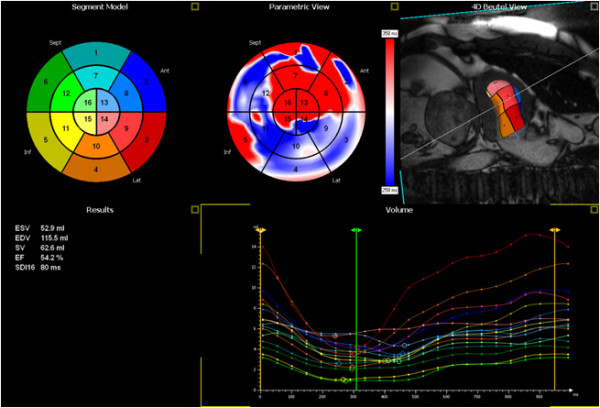
**Regional Volume measured by CMR_M; _16 segment model (top left) with corresponding LV shape (top right image), a static parametric view of the LV (top middle), global volume and EF values (bottom left) and time volume curves of all 16 segments (bottom right)**.

In a substudy (n = 10, all men, 66 ± 12 y) CMR data were also analyzed using the same center of mass-based method as 3DE (4D analysis MR CAP 1.0, Tomtec Gmbh, Unterschlessheim, Germany) for comparison with 3DE (Figure 3). First, the axes were arranged so the short axis views were parallel to each other and the long axis views were aligned in one axis. Frames for EDV and ESV measurement were identified in the same method as 3DE. Initial contours were set by tracing the endocardial borders in end-diastolic and -systolic images in 3 long axis slices (4-, 2- and 3-chamber views). Contour tracing was performed with automatic border detection and manual editing used as required for each step in the cardiac cycle and in the 3D model. Regional volumes (rEDV and rESV) were measured from the resulting volume.

### Time to Minimum Volume

Time to minimum volume was measured by both CMR and 3D techniques, which both had a temporal resolution of 20-25 Hz. The time taken to reach the minimal volume for each of the 16 segments was taken and visually checked from the time volume curves.

### Statistical analysis

Results for regional EDV and ESV are represented as mean and standard deviations. Correlations were performed between echo and CMR measurements, and the variation between the two measures was assessed using an F test, which compares the degree of variance between the methods. Agreement was expressed according to the method of Bland and Altman. A p value of < 0.05 was considered to be significant. Linear regression was used to find predictors of differences between change in 3DE and change in CMR for all LV parameters. Data analyses were performed using SPSS statistical software (SPSS v10, Chicago, IL).

## Results

### Patient characteristics

Of these 30 patients, 28 were men, and the mean age was 65 ± 11 years. All patients were referred for echocardiography and then subsequent CMR for LV evaluation at least 1 month after myocardial infarction. All patients had regional wall motion abnormalities prior to baseline scanning; most patients had an abnormality in the inferior wall (70%), followed by the anterior and posterolateral walls. Of patients who were revascularised between visits; 55% had coronary bypass grafting (with an average of 3.6 ± 1 grafts) and 6 patients had percutaneous transluminal coronary angioplasty while three patients received medical treatment. All patients underwent medical therapy or revascularization and were followed up over one year (follow-up scanning days, 3DE 357 ± 65; CMR 334 ± 54).

### Global Function at Baseline and at Follow-up

At baseline, 3DE underestimated CMR_L _measurements of EDV (173 ± 43 vs 197 ± 57 ml, r = 0.76, p < 0.01) and ESV (90 ± 38 vs 104 ± 54 ml, r = 0.87, p < 0.01), although EF was similar (51 ± 13 vs 49 ± 11%, r = 0.86 p < 0.01). Over 1 year follow-up all techniques showed a reduction of EDV and ESV and an increase in EF however this did not reach significance when comparing 3DE to the CMR_L _technique. For 3DE vs CMR_L _EDV (-6 ± 29 ml vs -5 ± 40 ml p = 0.08) and ESV (-5 ± 27 ml vs -3 ± 27 ml p = 0.09). Both methods showed improvement of EF 3DE 1 ± 9% vs CMR_L _1 ± 7% p = 0.09.

### Regional Volumes at Baseline and at Follow-up

Results of CMR and 3DE were analyzed in all 30 patients. Optimal images (< 2 segments not visualised) were obtained in 25 patients, but there were no significant differences in measures from the 5 patients did not have optimal images (> 2 segments not visualised). Although average baseline rEDV by the methods correlated (r = 0.37 p < 0.01), measures by CMR_L _were greater than for 3DE (13.04 ± 6 vs 8.21 ± 4 ml, p < 0.01). Baseline average rESV also correlated (r = 0.57, p < 0.01), but measures were also greater by CMR_L _than 3DE (6.89 ± 4 vs 4.37 ± 2 ml, p < 0.01).

CMR_L _and 3DE measures at follow-up showed similar correlations and differences between rEDV (r = 0.47, p < 0.01; 12.64 ± 6 vs 8.58 ± 4 ml, p < 0.01). Correlations between ESV were better (r = 0.61, p < 0.01), and follow-up CMR_L _ESV exceeded 3DE (6.87 ± 4 vs 4.82 ± 3 ml, p < 0.01).

With the change over time CMR_L _and 3DE measures at follow-up correlated poorly between rEDV (r = 0.03, p = 0.55; 0.40 ± 4 vs 0.37 ± 2 ml, p < 0.01) and between rESV (r = 0.07, p = 0.15 0.02 ± 2 vs 0.44 ± 1, p < 0.01).

Similar results were found when walls were separated into basal, mid and apical segments (Table 1) as well as coronary territories (Table 2). Although no subgroups provided good correlations between 3DE and CMR_L_, these were higher in non-revascularised segments than revascularised segments for both EDV (r = 0.37, p < 0.001 vs. 0.04, p = 0.51) and ESV (r = 0.22, p < 0.01 vs r = 0.02, p = 0.81), respectively.

**Table 1:** Regional left ventricular volumes were compared between visits when walls were separated into Basal, Mid and Apical segments (*p < 0.01)

	Basal	Mid	Apical
EDV (ml)	r = 0.33* F = 0.97, p = 0.45	r = 0.15, p = 0.04 F = 0.25*	r = 0.10, p = 0.27 F = 1.04, p = 0.42

ESV (ml)	r = 0.06, p = 0.41 F = 0.70, p = 0.03	r = -0.04, p = 0.64 F = 0.17*	r = -0.02, p = 0.83 F = 0.46*

EF (%)	r = 0.19, p = 0.01 F = 1.39, p = 0.04	r = 0.17, p = 0.02 F = 1.69*	r = 0.15, p = 0.09 F = 1.46, p = 0.02

**Table 2:** Regional left ventricular volumes were compared between visits when walls were separated into as well as coronary territories (*p < 0.01)

	RCA	LAD	LCx
EDV (ml)	r = -0.10, p = 0.37 F = 0.43*	r = 0.09, p = 0.13 F = 0.43*	r = -0.01, p = 0.92 F = 0.75, p = 0.06

ESV (ml)	r = -0.17, p = 0.10 F = 0.49*	r = 0.06, p = 0.36 F = 0.40*	r = -0.23, p = 0.01 F = 0.46*

EF (%)	r = 0.15, p = 0.17 F = 2.02*	r = 0.17, p = 0.01 F = 1.27*	r = 0.032, p = 0.73 F = 1.67*

### Comparison of Methods

CMR data was re-analyzed using the centre of mass method, so that is was now analogous with the technique for 3DE. The change in regional volume by 3DE and CMR_L _and 3DE and CMR_M _correlated poorly. For rEDV (3DE vs CMR_L _r = 0.03 p = 0.68, 0.11 ± 3 ml vs 0.12 ± 3 ml p = 0.94 and 3DE vs CMR_M _r = 0.04 p = 0.65, 0.26 ± 2 ml p = 0.69). Correlations between rESV were (3DE vs CMR_L _r = 0.03 p = 0.72, 0.27 ± 2 ml vs 0.36 ± 2 ml p = 0.70 and 3DE vs CMR_M _r = 0.07 p = 0.40, 0.05 ± 1 ml p = 0.31). Comparisons of individual segments at baseline and follow-up were similar to the comparison of average values. On a segmental basis, there was a high variation between regional volumes when LV walls were compared between visits with the exception of the anteroseptal wall.

### Assessment of Regional Time to Minimum Volume

3DE overestimated regional time to minimal volume at baseline and follow-up compared to both CMR methods (Table 3). There was only borderline correlation between both CMR methods and 3DE at baseline (r = 0.22, p = 0.05) and follow-up (r = 0.22, p = 0.06), however all stages showed large variation at each stage. There was no correlation and high variation when time to minimal volume was divided by the individual walls, basal, mid and apical segments or into coronary territories.

**Table 3:** Left ventricular time to minimum volume (*p < 0.01)

	3DE	CMR_L_	P	CMR_M_	p
Baseline	352 ± 61	-7 ± 89	r = 0.01, P = 0.86	-27 ± 96	r = 0.13, P = 0.11

Follow-up	372 ± 77	-38 ± 88	r = 0.22, P = 0.05	-30 ± 90	r = 0.22, P = 0.06

Change over time	20 ± 96	-31 ± 110	r = 0.15, P = 0.06	-3 ± 121	r = 0.12, P = 0.13

## Discussion

Although 3DE and CMR measures of global EDV and ESV correlate at baseline and follow-up, regional volumes correlate less well at both baseline and follow-up. Moreover, while the change in global volumes correlates well with each method, the change in regional volumes and time to minimum volumes correlates poorly.

### Global and regional volume calculations

In patients with myocardial infarction, the major source of error for echocardiographic measurement of global LV volumes derives from geometric assumptions, and for EF the problems derive from a combination of geometric assumptions and image processing [8]. Despite these errors global 3DE has been shown to correlate well to MRI both in cross-sectional studies [9-12] and over follow-up [5], although it underestimates volumes. This underestimation has been attributed to the spatial resolution of 3DE, which is insufficient to differentiate between the trabeculae and myocardial tissue [13]. Previous work has shown that not only do *global *3D volumes correlate well to MRI, but the change in volume correlates well, even in follow-up[5] The new finding in this paper is that the evolution of regional volumes correlates poorly. This may be due to the fact that standard software approaches to the segmental evaluation of both CMR_L _and 3DE are different. The floating-axis used by CMR_L _is based on first identifying important landmarks such as the LV apex, aortic valve, mitral annulus and RV insertions, whereas it is based on defining the center of mass by 3DE. The centre of mass may change as the LV remodels over time, whereas the CMR_L _axis is drawn from the annulus and apical plane, which are more likely to be stable over time. However, the limited correlation, even with the same method, suggests that the use of 3DE to track changes in regional volumes remains problematic. This may be due to the poor endocardial border definition and the ability for software to track over the cardiac cycle. Manual adjustments may help in the assessment of global volume assessment however adjusting over the cycle for assessment of regional volume may be hindered by this as it can cause an appearance of dyssynchrony.

A discrepancy is also shown by the comparison of the time to minimum volume for each technique. Both techniques have limited temporal resolution, and it is more difficult to mount an argument that CMR is the reference standard. However, the poor correlation of time to minimum volume between 3DE and both MRI techniques suggests that the methods should not be considered interchangeable.

### Validation of each technique

Good agreement has been documented between regional CMR volumes obtained with the CIM software and a segmental volume phantom [14]. This analysis also found agreement between two different points in time and by two independent observers.

Regional wall motion abnormalities have been visually assessed by 3DE [15,16], but this is dependent on the skill of the observer [17]. Although there have been several validation studies comparing 3DE volumes and EF to CMR [9,16,18,19], there has only been one that has quantitatively compared regional volumes [6]. This work used the same analysis package and did not compare regional volumes over time [6].

### Clinical implications

The assessment of regional volumes may permit analysis of the response of LV segments to interventions such as resynchronisation therapy [1]. A number of studies have shown that 3DE has overcome many of the limitations of two dimensional echocardiography with less test-retest variation, better reproducibility and accuracy in LV volume estimations [9,12,19]. However the latter data relate to global volumes - the results of this work emphasize that for regional volumes, the user should be aware of the method used by each technique to assess LV parameters, as these may differ between methods. The assessment of regional volumes by 3DE is poor in comparison with MRI using the same technique. This may be due to the technical limitations of 3DE, including artifacts and suboptimal image quality. Although global 3DE volumes compare well with CMR volumes, new developments in image quality and automated software will be needed before changes in regional volumes can be reliably followed with 3DE.

## Abbreviations

2DE: two-dimensional echocardiography; 3DE: three-dimensional echocardiography; CMR: cardiac magnetic resonance; CMR_L_: cardiac magnetic resonance landmark method; CMR_M_: cardiac magnetic resonance centre of mass method; EF: ejection Fraction; LVEDV: left ventricular end-diastolic volume; LVESV: left ventricular end-systolic volume; rEDV: regional end-diastolic volume; rESV: regional end-systolic volume

## Competing interests

The authors declare that they have no competing interests.

## Authors' contributions

CJ carried out the imaging, measurements of both echocardiograms and CMR, statistical analysis and drafted the manuscript. THM participate in the design, coordination of the study. All authors read and approved the final manuscript.
